# Giant Pericardial Tube Roll Aneurysm after the Treatment of Aortic Interruption

**DOI:** 10.1055/s-0040-1714122

**Published:** 2020-12-23

**Authors:** Mustafa O. Ulukan, Yahya Yildiz, Emin C. Ata, Didem M. Oztas, Korhan Erkanli, Murat Ugurlucan, Halil Turkoglu

**Affiliations:** 1 Department of Cardiovascular Surgery, Istanbul Medipol University Medical Faculty, Istanbul, Turkey; 2 Department of Anesthesiology and Reanimation, Istanbul Medipol University Medical Faculty, Istanbul, Turkey; 3 Department of Cardiovascular Surgery, Bagcilar Training and Research Hospital, Istanbul, Turkey

**Keywords:** interrupted aortic arch, congenital cardiac surgery, pericardial roll

## Abstract

Various techniques have been described for the treatment of interrupted aortic arch pathology. Graft interposition, either autologous or synthetic, is included among these methods. In this article, we present the images of giant pericardial roll aneurysm that was used for the treatment of aortic interruption during the newborn period.

Interrupted aortic arch is a rare congenital cardiac pathology that requires emergency repair during infancy. Different techniques, such as aortic mobilization and ascending aorta to descending aorta anastomosis, patch augmented anastomosis, or biologic or synthetic graft interposition, have been described in the literature for the treatment of this pathology. 1

The patient is a 3-year-old boy who underwent autologous pericardial tube graft interposition between the ascending and descending aorta for the treatment of Type B interrupted aortic arch during infancy. He had two insignificant apical muscular ventricular septal defects that did not require closure or pulmonary artery banding.

He has been followed periodically in the outpatient clinic, and the ventricular septal defects closed spontaneously. However, the pericardial roll dilated gradually. Originally it was 7 mm in diameter, and the size enlarged to 1.5 to 2.4 to 3.1 and finally 4.2 cm in diameter at 12, 18, 28, and 36 months, respectively.

Aneurysm formation was confirmed with computed tomography (CT) angiography that revealed a 4.2-cm dilatation of the pericardial tube ( Fig. 1A , B , Video 1 ). There was a stenosis visualized at the proximal anastomosis region. However, cardiac catheterization did not indicate a significant gradient ( Fig. 2A , B , Video 2 ).

**Fig. 1:**
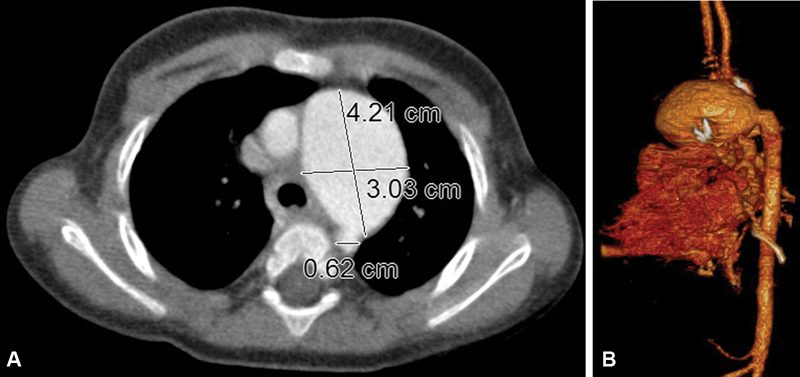
Axial ( **A** ) and 3-dimentional ( **B** ) computerized tomography angiography reveals dilatation of the pericardial tube.


**Video 1** Three-dimensional computerized tomography angiography video.


**Fig. 2:**
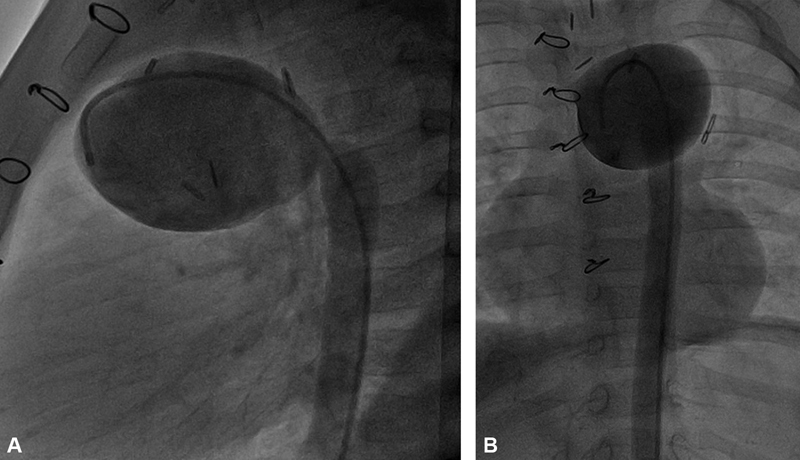
Preoperative cardiac catheterization: saggital ( **A** ) and frontal ( **B** ) view.


**Video 2** Cardiac catheterization video.


We performed surgical reconstruction of the aortic arch. The anterior wall of the aneurysm was resected leaving the posterior wall to enable growth. Reconstruction was done with a xenograft pericardial patch (Edwards Lifesciences, Irvine, CA). Operative and postoperative courses were uneventful. This patient was discharged on postoperative day 5. He remains asymptomatic, without evidence of further aortic dilatation for more than 12 months.

The reason for occurrence of the pericardial roll autograft aneurysm was attributed to the fresh use of the pericardial material and stenosis at the proximal anastomosis zone, that is, poststenotic dilatation. The advantages of pericardial roll bypass technique includes protecting the patient from negative effects of cardiopulmonary bypass, providing relatively shorter operation time and growth potential due to use of native tissue. However, while fresh materials may cause graft aneurysm, in the condition of longer gluteraldehyde fixation, calcification may occur and the growth potential may be compromised. Also, xenografts may be calcified. The appropriate fixation of the pericardium as a native tissue is beneficial to prevent complications. Complications including aneurysm, infection, or calcification have been reported in the literature by different authors following pericardial tube into position. 2 In complicated and life-threatening cases, resection of the pericardial materials and reconstruction of the remaining anatomy has been performed. 3 Autologous pericardial tube aneurysm after aortic interruption repair is possible but very rare. Treatment is required when symptoms of compression of the adjacent structures and progressive enlargement of the aneurysm occur.

As a limitation, we did not have a postoperative control CT, the patient was followed-up with echocardiography to prevent negative effects of CT such as radiation and contrast. If we had any doubts about gradient or dilatation, we were going to perform CT. But the patient was followed-up uneventfully.
